# *Drosophila* Nociceptors Mediate Larval Aversion to Dry Surface Environments Utilizing Both the Painless TRP Channel and the DEG/ENaC Subunit, PPK1

**DOI:** 10.1371/journal.pone.0032878

**Published:** 2012-03-05

**Authors:** Wayne A. Johnson, Justin W. Carder

**Affiliations:** Department of Molecular Physiology and Biophysics, Roy and Lucille J. Carver College of Medicine, University of Iowa, Iowa City, Iowa, United States of America; Duke University, United States of America

## Abstract

A subset of sensory neurons embedded within the *Drosophila* larval body wall have been characterized as high-threshold polymodal nociceptors capable of responding to noxious heat and noxious mechanical stimulation. They are also sensitized by UV-induced tissue damage leading to both thermal hyperalgesia and allodynia very similar to that observed in vertebrate nociceptors. We show that the class IV multiple-dendritic(mdIV) nociceptors are also required for a normal larval aversion to locomotion on to a dry surface environment. *Drosophila melanogaster* larvae are acutely susceptible to desiccation displaying a strong aversion to locomotion on dry surfaces severely limiting the distance of movement away from a moist food source. Transgenic inactivation of mdIV nociceptor neurons resulted in larvae moving inappropriately into regions of low humidity at the top of the vial reflected as an increased overall pupation height and larval desiccation. This larval lethal desiccation phenotype was not observed in wild-type controls and was completely suppressed by growth in conditions of high humidity. Transgenic hyperactivation of mdIV nociceptors caused a reciprocal hypersensitivity to dry surfaces resulting in drastically decreased pupation height but did not induce the writhing nocifensive response previously associated with mdIV nociceptor activation by noxious heat or harsh mechanical stimuli. Larvae carrying mutations in either the *Drosophila* TRP channel, Painless, or the degenerin/epithelial sodium channel subunit Pickpocket1(PPK1), both expressed in mdIV nociceptors, showed the same inappropriate increased pupation height and lethal desiccation observed with mdIV nociceptor inactivation. Larval aversion to dry surfaces appears to utilize the same or overlapping sensory transduction pathways activated by noxious heat and harsh mechanical stimulation but with strikingly different sensitivities and disparate physiological responses.

## Introduction

Evidence from both vertebrate and invertebrate models has contributed to a physiological and molecular description of how intense mechanical, thermal or chemical stimuli activate nociceptor neurons within the peripheral nervous system to produce the central sensation of pain [1], [2]. Many nociceptors can be classified into functionally and molecularly heterogeneous subtypes with abilities to detect specific and distinct noxious stimuli. However, a significant fraction of peripheral nociceptors are referred to as “polymodal” reflecting their expression of a collection of transduction molecules mediating activation by a variety of noxious sensory stimuli [1], [2].

In addition to their functional heterogeneity, nociceptors display an astounding plasticity with significant shifts in sensitivity when confronted with tissue injury, infection or inflammation [3]. Under normal circumstances, most nociceptors are prepared to respond to high threshold physical or noxious stimuli capable of acute tissue damage. However, tissue or nerve damage associated with injuries or inflammation can cause hypersensitization of nociceptors as part of a normal healing process [3], [4], [5]. Under these sensitized conditions, the nociceptor response to noxious stimuli can be greatly enhanced (hyperalgesia) or a pain response can be evoked by normally innocuous stimuli (allodynia). If hypersensitization does not resolve appropriately after an acute injury, beneficial pain can become pathological as a chronic and severely limiting problem. Precise molecular mechanisms responsible for these marked shifts in nociceptor sensitivity remain poorly understood. Therefore, the enhancement of nociceptor sensitivity associated with inflammatory pain continues to be a major clinical problem that impacts patient recovery and quality of life [1].

Characteriztion of genetic model organisms such as the fruit fly, *Drosophila melanogaster*, and the nematode, *C. elegans*, has contributed significantly to our understanding of a variety of sensory signaling processes. *Drosophila* larvae possess a complex peripheral nervous system capable of sensing a variety of environmental stimuli including odors [6], [7], light [8], temperature [9], [10], [11], [12], sound [13], [14], [15], [16], [17] and mechanical touch [18], [19]. Larvae display distinctive taxis behaviors in response to attractive or aversive stimuli that are mediated by many of the same sensory transduction channels and molecules implicated in vertebrate systems.

The *D. melanogaster* multidendritic or dendritic arborization neurons tile the inner surface of the larval epidermis with a complex dendritic arbor [20]. A subset of the multidendritic neurons referred to as the class IV(mdIV) neurons express both the *Drosophila* TRP channel, Painless(Pain) [21], and the *Drosophila* degenerin/epithelial sodium channel(DEG/ENaC) subunit, Pickpocket1(PPK1) [22], [23]. The mdIV neurons were previously characterized as nociceptive neurons capable of responding to either noxious heat(42°C) or noxious mechanical stimuli both resulting in a writhing “nocifensive” response [21], [24], [25]. The Pain TRP protein was shown to be required for the response to noxious heat and harsh mechanical stimulus [21]. The DEG/ENaC subunit PPK1 was required for the response to noxious mechanical stimulus [25]. However, the molecular mechanism of PPK1 activation and the endogenous function of mdIV neurons remain unclear. It has been proposed that the primary role of these nociceptors was to protect larvae from parasitoid wasps [24].

Recent studies have also shown that the larval mdIV neurons are sensitized by UV-induced tissue damage leading to both thermal hyperalgesia and allodynia [26]. This sensitization is dependent upon a *Drosophila* tumor necrosis factor(TNF) homolog, Eiger, that is released from apoptotic epidermal cells and the TNF receptor, Wengen, expressed on nociceptor neurons [26]. This exciting result provides additional evidence that the larval mdIV neurons display many of the same properties observed in mammalian nociceptors.

The *Drosophila* mdIV nociceptors share many morphological and functional characteristics with the multidendritic PVD neurons in the nematode, *C. elegans*, that have been functionally characterized for their role in behavioral responses to harsh touch and cold sensation [27], [28]. The nematode PVD neurons have been shown to require the TRPA-1 protein as a thermosensor for cold and the DEG/ENaC subunits MEC-10 and DEGT-1 for noxious mechanosensation [27]. Previous work in *C. elegans* has shown that members of the nematode DEG/ENaC family, MEC-4 and MEC-10, function as mechanotransducers in body touch neurons [29]. Vertebrate genomes express numerous **A**cid **S**ensing **I**on **C**hannels(ASICs) that are structurally related to the DEG/ENaC family but are proton-gated and appear to mediate acid-induced pain [30], [31]. Certain members of the ASIC family have also been associated with a mechanosensory function but have generated controversy due to tissue- and cell-specific functional differences [32], [33].

We have focused upon the larval mdIV neurons as an example of polymodal nociceptors mediating a broad variety of physiological responses including the previously demonstrated high threshold detection of noxious heat [21] and noxious mechanical [25] stimuli as well as mdIV nociceptor hypersensitization in response to UV-induced tissue damage [26]. However, these high threshold responses may not describe the true day-to-day role of these neurons in larval physiology. Recent studies characterizing the role of larval body wall neurons in control of larval locomotion have suggested that the mdIV neurons do not play a major role in the fundamental characteristics of locomotion such as coordination and stride length [34]. We show here that the same mdIV nociceptor neurons are required for larval aversion to locomotion into dry surface environments with a presumably distinct threshold for activation. These results suggest a more complex and versatile role for this single subtype of peripheral nociceptor than previously described. How does a single neuron respond to multiple types and intensities of activation with markedly different behavioral outputs? Our understanding of how similar or different these activation mechanisms may be should help to better understand the more subtle roles of polymodal nociceptors that are common in the human peripheral nervous system.

*Drosophila melanogaster* larvae are acutely sensitive to desiccation and remain associated with a moist food source until midway through the third larval instar when they exit the food source completely in search of an appropriate site for pupation [35], [36]. The choice of final pupation site can have a critical effect upon overall survival to adulthood since the immobilized pupa is exposed to a variety of potential environmental insults during morphogenesis. Pupation within the food source under moist conditions may result in lethality due to asphyxiation or a physical block of adult eclosion. Conversely, premature movement to an area of extreme low humidity prior to pupation could result in lethality due to desiccation [37], [38], [39], [40]. Previous studies have shown that pupation height within the microenvironment of a culture vial is dependent upon a number of environmental factors [41] including temperature [39], [40] and light [42], [43], [44]. However, three of the most influential factors are moisture content of the food media, external humidity and larval density [39], [41], [45], [46].

Each of these key environmental parameters may influence the same mechanism for modulation of pupation height. Larval locomotion after food exit requires a lubricating layer of moisture derived from the food source that is depleted as the distance away from the food source increases. The maximum distance of movement and maximum pupation height in traditional culture vials is, therefore, dependent upon the effective duration of the lubricating layer of moisture. This might be considered analogous to rubbing a wet finger across a sheet of dry glass where initial movement is easy until the layer of moisture is depleted causing increased friction and sticking. Wandering larvae display a remarkable ability to detect depletion of this transient lubrication layer resulting in an aversive response to dry surfaces that contributes to the overall choice of a pupation site. Further characterization of the molecular pathways responsible for the varied mdIV nociceptor response to distinct stimuli should yield information concerning developmental and functional modulation of nociceptor sensitivity.

## Results

### Larval pupation height is limited by a strong aversion to dry surface environments

**Figure 1:**
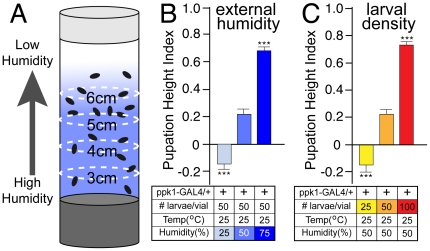
Control of pupation height behavior by the internal microenvironment of the culture vial. (A) Schematic representation of a *Drosophila* culture vial and the internal humidity gradient generated within the microenvironment of the plugged vial. Higher humidity at the bottom near the food surface is indicated as darker shading decreasing to low humidity at the top of the vial. Pupae are shown as black ovals within the designated zones indicated as height(cm) above the vial bottom used to calculate the pupation height index(PHI). PHI = ((#pupae>3 cm)-(#pupae<3 cm))/total # pupae. (BC) Pupation height behavior for ppk1-GAL4/+ control larvae represented by the indicated Pupation Height Index; (B) Pupation height is strongly influenced by external humidity. With constant larval density(50 larvae/vial), low external humidity(25%) causes larvae to pupate at sites lower in the vial indicated as a downward deflection. At higher humidity(50% and 75%), larvae choose to predominantly pupate at higher sites within the vial indicated as an upward deflection. (C) Pupation height is influenced by larval density. With vials grown at constant external humidity(50%), low larval density(25 larvae/vial) causes a low pupation height. Higher larval density causes a drastic increase in pupation height resulting from the altered microenvironment as larvae exit the food source. Error bars represent SEM with n≥15 for all values. ***P<.0001, one-way ANOVA with Tukey posttest.

Traditional larval culture vials with moist food at the bottom and a porous plug at the top create an internal microenvironment in which a humidity gradient is generated with a maximum at sites very close to the food surface decreasing to a minimum level at the top of the vial (Fig. 1A). The range of maximum and minimum points of the internal humidity gradient are determined by external humidity and food moisture content. When larval density is kept constant(50 larvae/vial), pupation height is strongly influenced by external humidity (Fig. 1B). This response was represented by a **P**upation **H**eight **I**ndex(PHI = ((#pupae>3 cm)-(#pupae<3 cm))/total # pupae) calculated using the final pupation position above or below a designated height in the vial(Fig. 1). In graphs of PHI, an upward deflection represents a tendency for pupation higher in the vial and a downward deflection, a preference for sites lower in the vial.

As demonstrated in previous studies [41], [45], [46], if external humidity is held constant, pupation height is also dependent upon larval density (Fig. 1C). Each of these environmental factors is able to influence the lubricating layer of moisture carried out of the food media by wandering larvae. Higher temperature and low humidity will both increase evaporation to limit the duration of the transient lubricating moisture layer. Higher larval density will result in increased liquefaction of food and increased amounts of food-derived moisture dragged up the side of the vial by wandering larvae. At higher larval density, the internal microenvironment of the vial is altered by coating of the vial sides by excessive moisture and food which alters the locomotion surface as well as shifting the internal humidity gradient such that larvae are able to reach higher levels for pupation (Fig. 1C).

### Larval aversion to dry surfaces is mediated by mdIV nociceptor neurons

**Figure 2:**
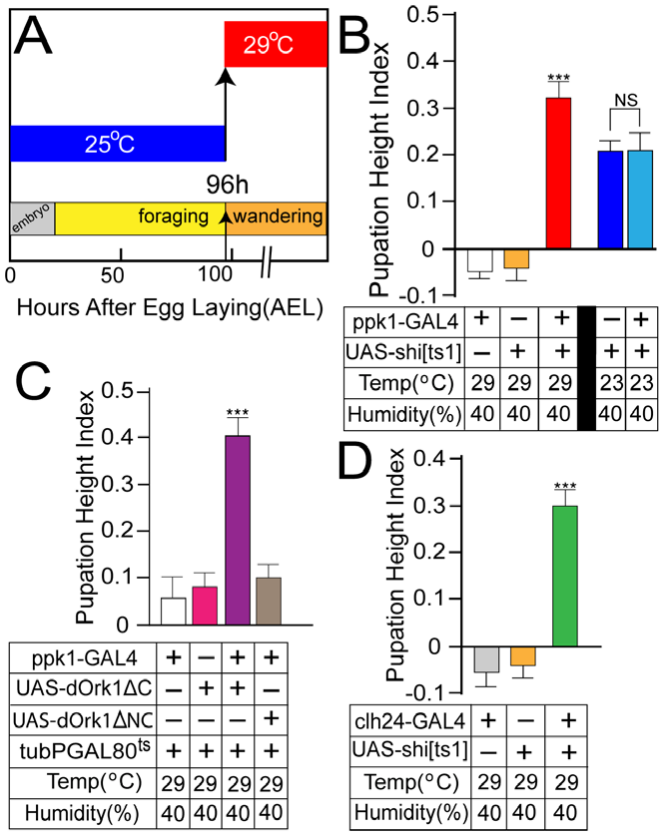
Pupation height selection is influenced by function of the mdIV nociceptor neurons. (A) Temperature shift protocol for stage-specific inactivation of mdIV nociceptors. Temporally staged larval collections(4 hr) were grown at 25°C throughout embryonic and larval foraging stages to maintain wild-type mdIV activity. At 96 h AEL, just as larvae are beginning wandering stage and food exit, cultures were shifted to 29°C to inactivate either Shi[ts1] or GAL80^ts^ respectively resulting in selective mdIV nociceptor inactivation during wandering stage. (B) mdIV nociceptor inactivation in *ppk1GAL4/UAS-shi[ts1]* larvae resulted in a significantly increased overall pupation height reflected as an upward deflection of the Pupation Height Index. Larval culture at 23°C to maintain Shi[ts1] function throughout pupation caused a higher overall PHI due to the lower temperature but resulted in no significant difference in PHI between control(*UAS-shi[ts1]/+*) and *ppk1-GAL4/UAS-shi[ts1]* larvae. (C) Electrical silencing of mdIV nociceptors in *tubPGAL80^ts^/+; ppk1GAL4/UAS- dOrk1ΔC* larvae also caused a shift to higher overall pupation height. Expression of dOrk1ΔNC, expressing an inactivated channel isoform, showed no significant difference in PHI from controls. (D) mdIV nociceptor inactivation in *clh24-GAL4/UAS-shi[ts1]* larvae resulted in the same significant upward deflection of the Pupation Height Index. The *clh24-GAL4* transposon is an independent GAL4 driver expressed specifically in the mdIV nociceptors similar to *ppk1-GAL4*. Error bars represent SEM with n≥15 for all values. ***P<.0001, one-way ANOVA with Tukey posttest.

Although previous studies have suggested that control of pupation height behavior is multigenic with effects linked to each of the three major chromosomes [38], [47], [48], no specific genes or regulatory mechanisms have been definitively identified. The role of the mdIV nociceptor neurons embedded within the larval body wall in control of pupation height was examined by neuron-specific manipulation of mdIV nociceptor activity in transgenic larvae. Temperature-sensitive isoforms of *shibire*(*shi[ts1]*) and GAL80^ts^ were used with a *ppk1-GAL4* transgene to specifically manipulate mdIV nociceptor function only after 96 h of development when larvae normally begin to exit the food source and display wandering behavior (Fig. 2A). The *UAS-shi[ts1]* transgene encodes a *Drosophila* dynamin homologue necessary for synaptic vesicle recycling that is inactivated at restrictive temperatures >29°C [49]. Larvae derived from four hour egg collections were placed in vials at constant density(50 L/vial) and allowed to grow at 25°C until 96 h AEL when vials were shifted to 29°C (Fig. 2A). Inactivation of mdIV neurons in *ppk1-GAL4/UAS-shi[ts1]* larvae caused a striking shift of pupation site selection to higher regions of the vial (Fig. 2B). Larvae of the same genotypes grown throughout development at 23°C to prevent Shi[ts1] inactivation showed an overall higher PHI in both control and expermental vials due to the cooler temperature (Fig. 2B). However, there was no significant difference from control vials consistent with this shift in PHI being caused by mdIV nociceptor inactivation.

Similar experiments using the constitutively active dOrk1ΔC [50] channel to electrically silence mdIV neurons yielded very similar results consistent with a shift to higher pupation heights(Fig. 2C). In these experiments, all larvae carried the *tubPGAL80^ts^* transposon to ubiquitously suppress GAL4 function at 25°C. The same temperature shift protocol (Fig. 2A) was used to inactivate GAL80^ts^ at 96 h AEL by shifting cultures to 29°C allowing subsquent GAL4-dependent expression. Larvae expressing the dOrk1ΔNC isoform containing a channel inactivating mutation [50] showed no significant difference from controls These results suggested that the mdIV nociceptors were playing a key role in the larval aversion to high friction locomotion on dry surfaces.

mdIV nociceptors were also inactivated using the alternative GAL4 driver transposon, *clh24-GAL4*, expressed in an mdIV-specific pattern essentially identical to *ppk1-GAL4* [34]. Transgenic *clh24-GAL4/UAS-shi[ts1]* larvae showed the same significant shift to a higher PHI when assayed in the same temperature shift protocol. These results demonstrate that the observed shift in pupation height behavior is not due to an artifact associated with use of the *ppk1-GAL4* driver transposon.

**Figure 3:**
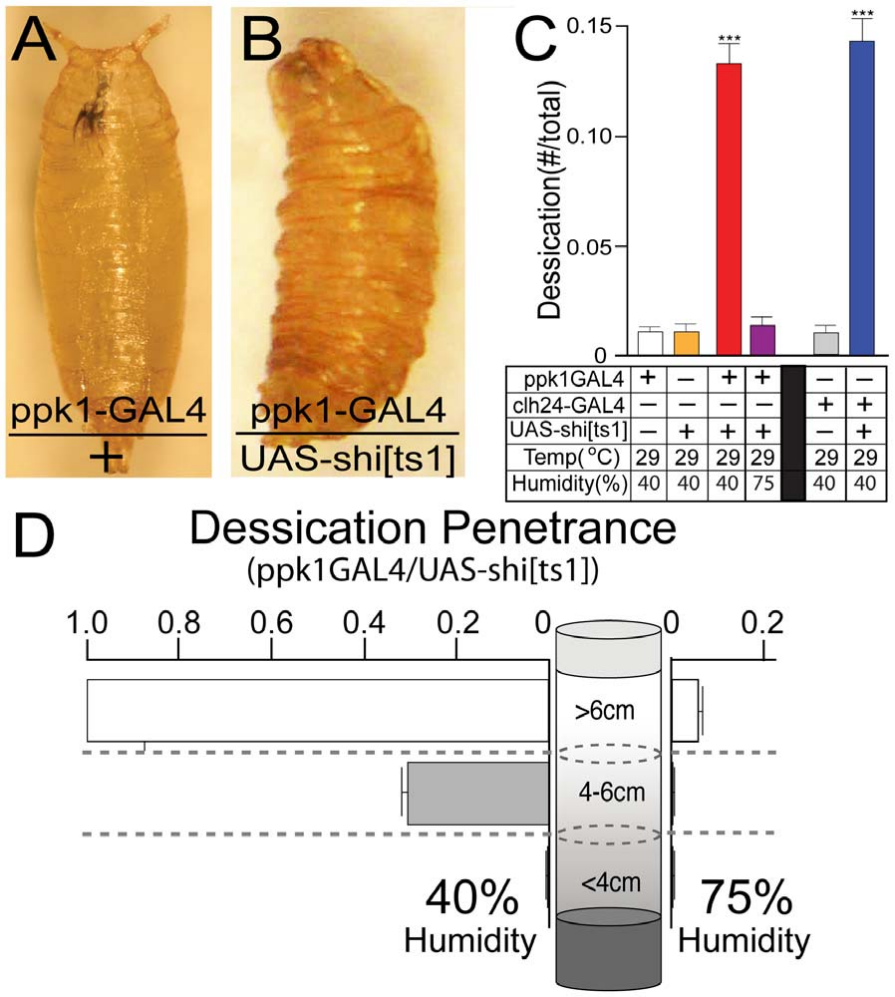
Transgenic mdIV nociceptor inactivation caused a lethal desiccation phenotype in larvae moving inappropriately into higher regions of the culture vial. (AB) Lethal larval/pupal desiccation phenotype resulting from mdIV nociceptor inactivation. (A) Wild-type pupa. (B) Representative desiccated *ppk1-GAL4/UAS-shi[ts1]* pupa recovered from high region of vial(>7 cm). (C) mdIV nociceptor inactivation in *ppk1GAL4/UAS-shi[ts1]* larvae caused accumulation of desiccated larvae stranded in upper regions of the vial. The increase in desiccated larvae was completely suppressed by growth at high external humidity(75%). mdIV nociceptor inactivation using the alternative *clh24-GAL4* mdIV-specific driver caused the same lethal desiccation phenotype. (D) Penetrance of lethal desiccation phenotype in *ppk1-GAL4/UAS-shi[ts1]* larvae relative to pupation height. Lethality by desiccation is 100% in low humidity regions above 6 cm but is never seen below 4 cm near the moist food source. Culture growth at high external humidity(75%) resulted in nearly complete suppression of the lethal desiccation phenotype. Error bars represent SEM with n>15 for all values. ***P<.0001, one-way ANOVA with Tukey posttest.

In addition to the upward shift in pupation height, mdIV nociceptor inactivation caused the increased appearance of larval lethality at sites near the top of the vial (Fig. 3). At 40% external humidity, all larvae reaching pupation heights >6 cm displayed a lethal desiccation phenotype in which larvae appeared to desiccate and die prior to normal pupariation (Fig. 3A, 3B). The number of desiccated larvae was increased more than 15× in *ppk1-GAL4/UAS-shi[ts1]* larvae relative to controls and this was completely suppressed by growth at higher external humidity(75%) (Fig. 3C). In contrast, larval desiccation was never seen at levels <4 cm where humidity is at its highest and was suppressed by growth at 75% external humidity (Fig. 3D). The same increase in lethality by desiccation near the top of the vial was observed with *clh24GAL4/UAS-shi[ts1]* larvae utilizing the alternative mdIV-specific clh24-GAL4 driver transposon [34]. These results suggested that mdIV nociceptor inactivation caused a failure to sense movement onto drier surfaces resulting in stranding and lethal desiccation.

### Transgenic hyperactivation of larval mdIV nociceptor neurons causes hypersensitivity to dry surfaces

**Figure 4:**
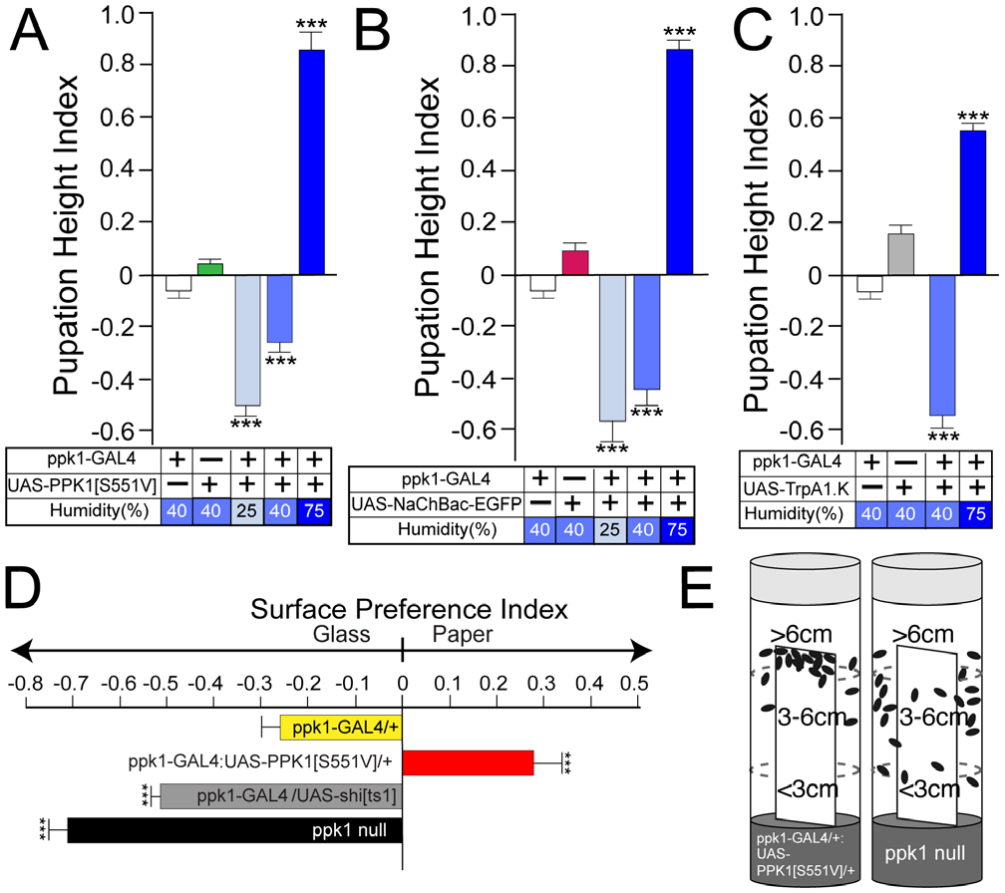
Hypersensitive aversion to dry surfaces caused by transgenic hyperactivation of mdIV nociceptor neurons. (A) Hyperactivation of mdIV nociceptors by transgenic expression of the constitutively-active PPK1[S551V] isoform resulted in a drastic decrease in pupation height depicted as a downward deflection of Pupation Height Index. This hypersensitive aversion behavior was completely suppressed by high external humidity(75%). (B) Hyperactivation of mdIV nociceptors by transgenic expression of the low-threshold voltage-gated Na channel *UAS-NaChBac-EGFP* caused a similar decrease in pupation height that was suppressed by high external humidity(75%). (C) Hyperactivation of mdIV nociceptors by transgenic expression of the heat-activated TrpA1.K channel caused the same significant decrease in overall pupation height that was suppressed by growth at high humidity(75%). All vials contained 50 larvae. (DE) Larval surface preference assay. (D) Third-instar larvae of the indicated genotypes were given a choice for pupation on the glass vial sides or on a strip of wetted Whatman filter paper inserted vertically at the center of the vial(see Materials and Methods). A preference index was calculated to indicate a preference for pupation on the moistened filter paper as a deflection to the right and a preference for the glass surface as a deflection to the left. Larvae with hyperactivated mdIV nociceptors expressing the constitutively-active PPK1[S551V] isoform showed a strong preference for pupation on the moist filter paper surface. Inactivation of mdIV nociceptors in ppk1GAL4/UAS-shi[ts1] and *ppk1* null larvae caused an enhanced preference for pupation on the glass surface. (E) Schematic representation of the larval surface preference assay. Error bars represent SEM with n≥15 for all values. ***P<.0001, one-way ANOVA with Tukey posttest.

Reciprocal hyperactivation of mdIV nociceptors caused a hypersensitivity to dry surfaces resulting in a significantly lower pupation height (Fig. 4). Very similar results were obtained by transgenic expression of three different modifiers of neuronal activity suggesting that this phenotype was not the result of insertional artifacts or aberrant transposon interactions. Neuron-specific expression of the constitutively-active PPK1[S551V] isoform [51] in mdIV neurons caused a drastic shift to a lower pupation height (Fig. 4A). Imaging of mdIV nociceptor neurons coexpressing UAS-PPK1[S551V] and UAS-CD8GFP detected no gross morphological or developmental abnormalities(data not shown) consistent with this behavioral response resulting from alterations in mdIV neuronal activity. This preference for pupation sites very near to the food surface where humidity is at a maximum was suppressed by growth at high humidity(75%) demonstrating that the lower pupation height was not due to a physical inability to climb the side of the vial. Essentially identical results were obtained by mdIV hyperactivation using either the low-threshold voltage-gated sodium channel, *NaChBac-EGFP* [52] (Fig. 4B) or the temperature-activated TrpA1.K channel subunit [53] (Fig. 4C).

Hypersensitization of larval aversion to dry surfaces was also demonstrated using a surface choice assay in which wandering larvae were given the opportunity to choose between a strip of wetted Whatman filter paper inserted vertically into the center of the vial and the glass surface of the culture vial (Fig. 4D, 4E). The larval choice between surfaces takes place primarily at the food surface immediately following food exit. Individual larvae will often exit and reenter the food source multiple times before committing to stay on the glass surface. Although a small fraction of the larvae will move farther up the surface before returning to the food, the vast majority make their decision very close to the food surface. This assay is a simple one that depicts a simple choice for larvae whether to stay on that surface or return to the food source. The relative preference for glass vs. wetted filter paper was represented as a surface preference index(see Experimental Procedures) and depicted as a positive/right deflection indicating preference for pupation on wetted paper surface (Fig. 4D). Under the conditions for this assay, wild-type controls(*ppk1-GAL4/+*) displayed a weak preference for the glass surface while larvae expressing the constitutively-active PPK1[S551V] isoform in mdIV neurons displayed a strong preference for pupation on the wetted filter paper over the dry glass surface indicated as a deflection of the surface preference index to the right (Fig. 4D). In contrast, both the mdIV-inactivated *ppk1-GAL4/UAS-shi[ts1]* and *ppk1* null mutant larvae showed a strong suppression of aversion to the dry surface indicated as an increased deflection to the left relative to the *ppk1-GAL4/+* control (Fig. 4D).

In addition to the marked shift in preference for the wetted surface, mdIV nociceptor hyperactivated *ppk1-GAL4:UAS-PPK1[S551V]* larvae showed a very interesting pattern of pupation at the top most end of the filter paper (Fig. 4E). Hyperactivated larvae, (>99%) choosing the wetted filter paper, climb to the very top edge of the filter paper where they pupate bunched together within the top 0.5 cm. This observation indicates that the low pupation height of mdIV nociceptor hyperactivated larvae in traditional glass vials is not due to a physical defect in locomotor activity making it impossible to climb the sides of the vial. When presented with an appropriate low-friction surface, either at high humidity or with the wetted filter paper, larvae are able to continue climbing until the top of the surface is reached. This result also suggests that the observed changes in pupation height are not due to changes in larval photokinesis. When grown under the same light conditions, larvae with hyperactivated mdIV nociceptor neurons will climb to the top of the wetted filter paper suggesting that their low pupation height on the glass surface is not due to a hypersensitivity to light.

Previous work has shown that mdIV nociceptor activation by either noxious heat or noxious mechanical stimuli elicits a vigorous twisting escape behavior referred to as a “nocifensive” response [21], [25]. Although transgenic hyperactivation of mdIV nociceptors caused a marked change in pupation height behavior, we did not observe any evidence of the twisting escape behavior previously associated with mdIV activation. This observation suggests that these two physiological outputs of mdIV nociceptor activation utilize strikingly different activation thresholds or distinct sensory transduction pathways. Alternatively, neuronal circuitry may act to suppress the writhing response during locomotion.

### mdIV nociceptor function in dry surface aversion behavior requires both the Painless TRP channel and the DEG/ENaC subunit, Pickpocket1

**Figure 5:**
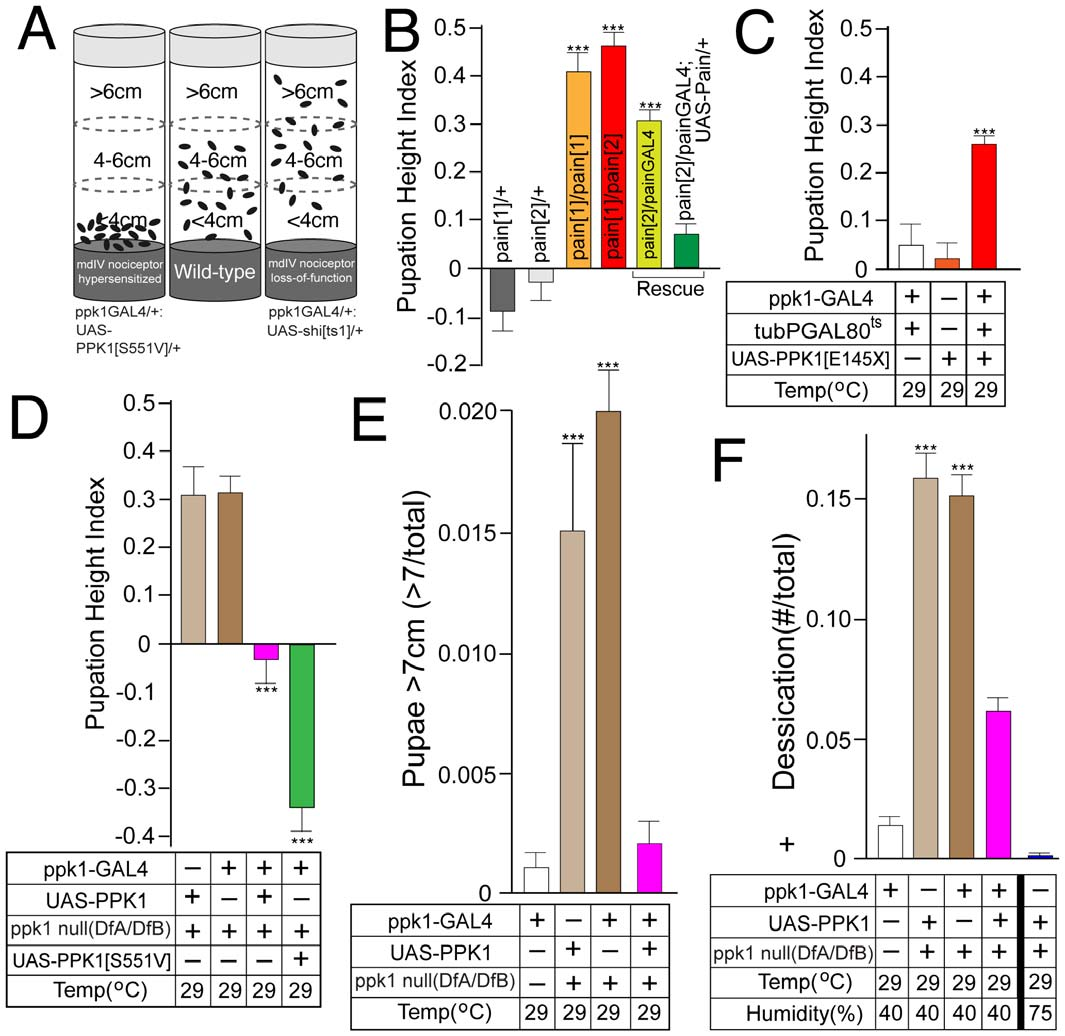
Larval aversion to dry surfaces requires both the DEG/ENaC subunit PPK1 and the TRP channel Pain. (A) Schematic depiction summarizing opposing mdIV nociceptor gain-of-function and loss-of-function phenotypes. (B) *pain* mutant alleles showed increased pupation height phenotype that was rescued by transgenic expression of wild-type Pain protein. (C) Transgenic expression of the dominant-negative PPK1[E145X] isoform after 96 h AEL caused an increase in pupation height similar to that observed with mdIV nociceptor inactivation. (D) *ppk1* null mutant larvae displayed an increase in pupation height that was rescued by transgenic expression of wild-type PPK1 or the constitutively-active PPK1[S551V] isoform in mdIV nociceptor neurons. (E) Fraction of total larvae pupating in the top regions of the vial(>7 cm) was significantly increased in *ppk1* null mutants and rescued by mdIV-specific expression of wild-type PPK1. (F) Fraction of total larvae displaying the lethal desiccated larval phenotype was significantly increased in *ppk1* null mutant larvae. Larval desiccation was rescued by transgenic expression of wild-type PPK1 or by growth at high humidity(75%). All vials were shifted to 29°C/40% humidity at 96 h AEL unless otherwise indicated. Error bars represent SEM with n≥15 for all values. ***P<.0001, one-way ANOVA with Tukey posttest.

Analysis of the effect of mdIV nociceptor function on larval pupation height provided a striking contrast between gain-of-function/hypersensitized and loss-of-function/inactivated phenotypes (Fig. 5A). Larvae expressing the constitutively-active PPK1[S551V] isoform displayed a low pupation height phenotype in direct contrast to the increased pupation height seen in mdIV-inactivated animals. Results from the surface preference assay (Fig. 4D, 4E) suggested that *ppk1* null mutant larvae displayed a loss-of-function phenotype very similar to mdIV inactivated *ppk1GAL4/UAS-shi[ts1]* larvae (Fig. 4D).

The TRP channel, Pain, is expressed in mdIV nociceptors and is required for the larval response to noxious heat and harsh mechanical stimulus [21]. The DEG/ENaC subunit, PPK1, is specifically expressed in the mdIV nociceptor neurons and has been shown to be required for the response to noxious mechanical stimulus [25]. We have also demonstrated a role for PPK1 in early foraging stages [51]. Based upon these previously characterized roles for Pain and PPK1 in mdIV nociceptor function, we asked whether they were also required for the mdIV-dependent pupation height phenotype.

Previously characterized *pain* loss-of-function alleles [21] showed a significantly increased pupation height (Fig. 5B) comparable to that seen in mdIV nociceptor inactivated larvae (Fig. 2B, 2C). This was consistent for multiple *pain* insertional mutant alleles and was rescued by transgenic expression of wild-type Pain protein under the control of the *painGAL4* transposon, itself a weak insertional *pain* mutant (Fig. 5B). These results suggested that the mdIV nociceptor mechanisms mediating physiologically relevant larval pupation height selection may utilize the same or overlapping processes previously characterized for the larval response to noxious heat and harsh mechanical stimulus [21].

The role of the DEG/ENaC subunit, PPK1 in this behavior was examined using the same assay for pupation height. Expression of the previously characterized dominant-negative isoform PPK1[E145X] [23] was induced in *tubPGAL80^ts^;ppk1-GAL4/UAS-PPK1[E145X]* larvae at 96 h AEL by shifting the incubation temperature to 29°C resulting in a comparable increase in pupation height (Fig. 5C). Null mutant *ppk1* larvae were generated using a previously characterized pair of overlapping deficiency chromosomes(DfA/DfB; see Experimental Procedures) that selectively remove only *ppk1* [51]. *ppk1* null mutant larvae displayed a comparable increase in pupation height that was completely rescued by transgenic expression of wild-type PPK1 or PPK1[S551V] under control of the *ppk1-GAL4* transposon (Fig. 5D).

In addition to the increased pupation height, the appearance of desiccated *ppk1* null mutant larvae near the top of the vial was even more pronounced than that seen in mdIV nociceptor inactivated larvae (Fig. 5E, 5F). This is easily represented by plotting simply the number of larvae pupating above 7 cm at the top of the vial indicating that 15–20% of *ppk1* null mutant larvae will move inappropriately into the drier regions at the top of the vial relative to ≤1% of wild-type(*ppk1-GAL4/+*) larvae (Fig. 5E). This phenotype was completely rescued by transgenic expression of wild-type PPK1 under control of the *ppk1-GAL4* transposon. Essentially all *ppk1* null mutant larvae pupating in the higher regions of the vial displayed the same lethal desiccation phenotype seen with mdIV nociceptor inactivated larvae (Fig. 5F). The desiccated phenotype was rescued by transgenic expression of wild-type PPK1 or by growing cultures at 75% humidity (Fig. 5F).

**Figure 6:**
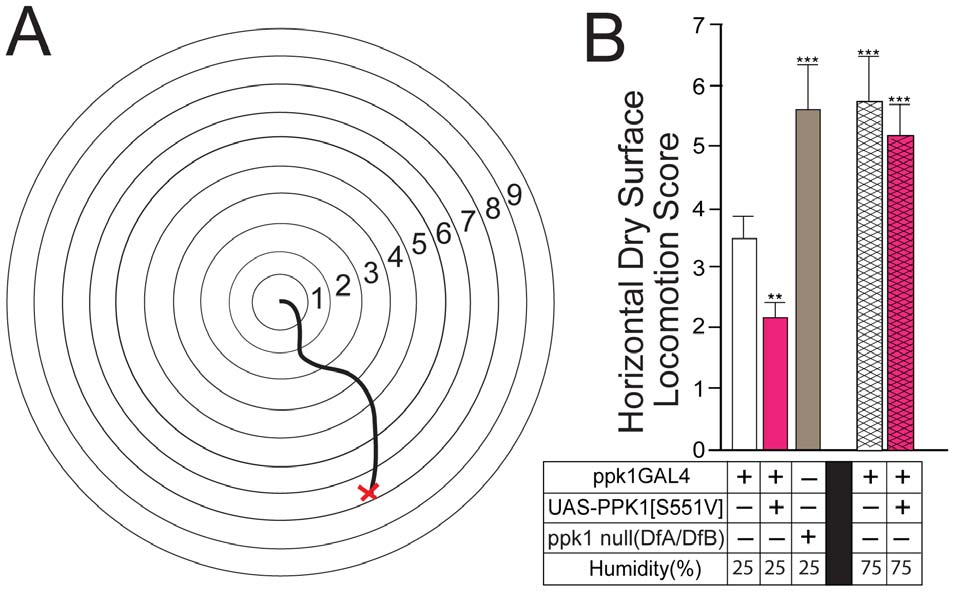
Correlation of genotype-specific effects of transgenic mdIV nociceptor manipulation in a horizontal dry surface locomotion assay. (A) Schematic representation of the horizontal dry surface locomotion assay(see Materials and Methods). (B) Horizontal locomotion behavior parallels genotypic differences in PHI. Wandering stage larvae of the indicated genotypes were assayed in an alternative protocol for larval locomotion on a dry horizontal glass surface under constant humidity. Higher scores represent increased distance of movement away from a moist filter over a dry glass surface(see Materials and Methods). Paralleling results from determination of PHI, *ppk1-GAL4/UAS-PPK1[S551V]* larvae expressing a constitutively active PPK1 isoform preferred to avoid extensive locomotion over the dry surface. The wild-type control *ppk1-GAL4/+* larvae moved an intermediate distance and *ppk1* null mutant larvae(w^1118^; DfA/DfB) moved a significantly increased distance over the dry glass surface before becoming stranded. When assayed under the same conditions except at high humidity levels(75%), both *ppk1-GAL4/UAS-PPK1[S551V]* and *ppk1-GAL4/+* larvae displayed significantly increased locomotion over the dry glass surface. Error bars represent SEM with n>15 for all values. **P<.01, ***P<.0001, one-way ANOVA with Tukey posttest.

Results presented thus far support the suggestion that the mdIV nociceptor neurons embedded in the larval body wall may function to detect increased frictional drag as larvae exit the food source and attempt to crawl on the dry glass walls of the culture vial. However, as described earlier, larval food exit and choice of pupation site is a complex behavior dependent upon a variety of developmental and physiological factors including characteristics of the microenvironment within the culture vial. We utilized a simple larval locomotion behavior observed on a dry flat glass surface under constant humidity to further isolate and define these many factors determining larval aversion to movement onto dry surfaces (see Materials and Methods). Wandering larvae of the appropriate genotypes were placed on a wetted filter (0.5 mm diameter, 5 larvae/filter) at the center of a flat dry glass sheet under constant controlled humidity, temperature and light (see Materials and Methods; Fig. 6A). A grid of concentric circles was placed beneath the glass sheet to allow scoring of larval movement away from the wetted filter and onto the dry glass surface. Larvae were allowed to crawl for 10 min under these conditions after which they were individually scored for their position within the grid of concentric circles. Therefore, a higher numerical score indicates increased distance of locomotion away from the wetted filter onto the dry glass surface.

Results from analysis of key genotypes previously characterized in the pupation height assay showed a remarkably close correlation (Fig. 6B). When assayed at low humidity(25%), *ppk1-GAL4/UAS-PPK1[S551V]* larvae showed limited movement onto the dry surface (Fig. 6B) consistent with their previously demonstrated low pupation height (Fig. 4A). This was significantly less than *ppk1-GAL4/+* control larvae which moved an intermediate distance onto the dry surface (Fig. 6B) consistent with their previously demonstrated intermediate PHI (Fig. 2B). ppk1 null mutant larvae moved a significantly higher distance over the dry glass surface before becoming stranded (Fig. 6B). This extended locomotion on the dry surface is consistent with the observed tendency of ppk1 null mutant larvae to move innappropriately into higher regions of the culture vial where they subject to stranding and lethal desiccation (Figs. 2 and 3). When the same genotypes were assayed at high humidity (75%), both *ppk1-GAL4/UAS-PPK1[S551V]* and *ppk1-GAL4/+* larvae displayed locomotion distances comparable to *ppk1* null mutants (Fig. 6B).

The close correlation of relative results from both the pupation height assay and the horizontal dry surface locomotion assay suggest that the observed differences in pupation height displayed by mdIV nocicpetor activity mutants within the culture vial is not determined primarily by antigeotactic or phototactic behavior. In addition, larval movement appears to not rely upon specific response to the humidity gradient. Retention of the relative behavioral scores of control, hyperactivation and inactivation mutant larvae in the horizontal dry surface assay is consistent with our hypothesis that the mdIV nociceptor neurons are responding to frictional drag as larvae deplete their lubricating layer of moisture with increased movement across the dry glass surface.

## Discussion

### Mechanosensation vs hygrosensation vs nociception

The efficient ability of higher organisms to monitor their external environment is based upon not only the diversity of external sensory organ types and their unique modalities but also the ability of individual neurons to respond to multiple types of stimuli with profound plasticity in response thresholds [3], [54], [55]. A more extensive molecular understanding of these characteristics may help to direct studies toward new clinical approaches for alleviation of pain that could reduce side-effects and potential for abuse. An expanded understanding may require a broadened definition of the concept of a “nociceptor” as part of the larger process of somatosensory signaling. Therefore, to understand the complexity of somatosensory signaling at the cellular level, we must ask when and how does the transition take place between mechanosensation and nociception or between thermosensation and pain induced by noxious heat.

The *Drosophila* larval mdIV nociceptors represent an excellent genetic model for experiments to address these key questions. The mdIV neurons have previously been associated with a nociceptive function demonstrated by their responses to noxious heat and harsh mechanical stimuli [21], [25] requiring the TRP channel Pain and the DEG/ENaC subunit PPK1. Results presented here demonstrate an essential role for the mdIV nociceptor neurons in larval aversion to dry surface environments manifested as a choice in relative pupation height. Wild-type *D. melanogaster* larvae display the ability to monitor moisture levels as they move across surfaces allowing a choice to return to the food source or lower regions of the vial where humidity and moisture is highest. This may serve to limit or prevent frictional wounding of the epidermis caused by contact with the dry surface. This aversion behavior requires function of both the Painless TRP channel and the PPK1 DEG/ENaC subunit.

Larval monitoring of external humidity could be accomplished through a few different but potentially complementary physiological mechanisms. Activity of mdIV nociceptors could function in the direct mechanosensory detection of changes in larval hemolymph internal hydrostatic pressure in response to evaporative moisture loss. Numerous studies in adult arthropods have suggested that sensory neurons in the adult antenna respond to increased or decreased external humidity through a direct mechanosensory function [56], [57], [58], [59]. In *Drosophila* adults, the TRP channel family members, *water witch* and *nanchung* have been demonstrated to play a role in hygrosensation presumably through a mechanosensory function [60]. During larval stages, although the external cuticle serves an important protective function as larvae maneuver through their environment, the cuticle is highly permeable to moisture such that exposure to low humidity can cause a loss of internal water content by evaporation and a decrease in internal hydrostatic pressure. The morphological positioning of the mdIV dendritic arbors within the body wall would be entirely consistent with a function to directly detect internal pressure changes as a mechanosensory stimulus.

Alternatively, larvae may detect dry environments indirectly due to the more rapid evaporation of the lubricating layer of moisture necessary for efficient locomotion. One potential hypothesis could describe the detection of increased frictional drag to limit larval movement or possibly cause frictional wounding of the larval epidermis. Either of these processes could be consistent with the behavioral results observed for final pupation height and are not necessarily exclusive. Although these seemingly very distinct types of stimuli would appear to have quite unique activation thresholds, it can often be difficult to definitively separate sensory modalities within a single polymodal sensory neuron. Clear boundaries between mechanosensation, hygrosensation and nociception may be quite blurred at a molecular level since the behavioral endpoints may depend upon complementary peripheral inputs to a central behavioral circuit. The question of how a single nociceptor neuron might utilize the same signal transducing ion channels to respond to such strikingly different stimuli is of great relevance.

Published studies demonstrate that stimulation of mdIV nociceptors by noxious heat (42°C) or harsh mechanical stimuli results in a violent writhing escape behavior described as a “nocifensive” response [21], [26]. In contrast, our results demonstrate that hypersensitization or activation of mdIV nociceptors by transgenic expression of three different activating ion channels (Fig. 3) caused a significant shift in pupation height behavior but did not result in a direct induction of the described “nocifensive” behavior.

This striking difference in behavioral response to activation of the same neurons could be explained by two broad alternative models describing either a mechanism that is exogenous to the mdIV nociceptors themselves or one involving endogenous changes in the mdIV nociceptors. Both models incorporate concepts established for central and peripheral pain sensitization in vertebrates [61], [62].

The more intensive response to noxious heat, although initiated by and dependent upon mdIV nociceptor activation, could result from the recruitment of additional firing units either peripherally or centrally to drive a massive physical response [1], [61]. A higher frequency of action potentials resulting from a strong stimulation by noxious heat could result in an expanded central activation of downstream neurons to cause a more vigorous behavioral response. This type of recruitment is thought to occur in vertebrates through central disinhibition of inhibitory interneurons allowing nonnociceptive afferents to influence pain transmission circuitry [62]. This mechanism for central pain sensitization allows normally innocuous stimuli to cause enhanced pain. Little information is currently available for invertebrate model organisms concerning central circuits mediating the pain response.

Alternatively, the difference in behavioral response to mdIV nociceptor activation could result from a peripheral sensitization due to molecular and biophysical alterations within the mdIV neurons themselves. We have shown that mdIV-specific expression of the constitutively active PPK1[S551V] isoform alone was capable of hypersensitization of the mdIV nociceptor behavioral response. This suggests that changes in expression or activity levels for key mediators of the mdIV response could cause marked alterations in behavioral response. Published studies have also shown that the mdIV nociceptors are capable of hypersensitization in response to endogenous cytokines [26] analogous to those involved in vertebrate nociceptor sensitization during inflammation [63], [64], [65]. The observed allodynia resulting from UV-induced tissue damage in *Drosophila* larvae was dependent upon a tumor necrosis factor(TNF) homolog, Eiger, that is released from damaged epidermal cells [26]. These results suggest a hypothesis in which mdIV nociceptor activation during high friction locomotion could result from the release of inflammatory mediators such as Eiger from epidermal cells damaged by frictional wounding. This would be consistent with numerous examples of inflammatory mediators released from damaged keratinocytes that are capable of nociceptor sensitization in vertebrates [5].

A peripheral sensitization model explaining the range of mdIV-dependent responses would require a striking transformation from an essentially innocuous mechanosensory response resulting from altered frictional drag during locomotion to a massive writhing whole body response to noxious heat. This shift in functional sensitivity observed in the mdIV nociceptor also represents an excellent genetic model for examination of the widely seen phenomenon of multiple sensory modalities associated with a single sensory neuron. We have demonstrated that the complex behavioral process for pupation site selection requires both the Pain TRP channel and the PPK1 DEG/ENaC subunit. Although there are numerous examples in both vertebrate and invertebrate models of individual nociceptors expressing both TRP and DEG/ENaC/ASIC ion channels [25], [27], [66], [67], [68], [69], the functional interaction between these two channel subtypes is not well understood. No direct interactions between channel subtypes within the same neuron have been demonstrated and numerous studies suggest that they function relatively independently to sense thermal and mechanical stimuli using distinctly different molecular components [27].

The demonstrated role for both Pain and PPK1 in the determination of responses to changing environmental surfaces does not necessarily imply identical or overlapping functions. The complexity of the pupation height behavioral paradigm may allow unique but complementary contributions from each channel subtype resulting in the overall phenotypic outcome. Alternatively, the interaction could be indirect through an overall effect upon neuronal excitability. Previous work has shown that TRPA1 may play an essential but indirect role in nociceptor sensitization in response to endogenous compounds associated with inflammation and edema acting as proalgesic agents to elicit inflammatory pain [70], [71], [72]. Our ability to efficiently modify mdIV nociceptor activity and to alter mdIV-specific patterns of gene expression should allow a genetic analysis of polymodal nociceptor function and the individual gene networks responsible for eliciting a range of responses to nociceptor activation.

## Materials and Methods

### Drosophila stocks and reagents

Fly stocks were raised on standard cornmeal-yeast-agar medium. Crosses were performed at 25°C at 50% humidity unless stipulated in assay procedures below. *UAS-ORK1ΔC, UAS-ORK1ΔNC, UAS-NaChBac-EGFP, tubPGAL80^ts^* and *UAS-TrpA1.K* stocks were obtained from the Bloomington Drosophila Stock Center. We thank Toshi Kitamoto for *UAS-shi[ts1]* stocks and J.B. Thomas for *clh24-GAL4* transgenic stocks.

The *ppk1* null mutant genotype was produced using two previously described overlapping deficiency chromosomes [51] generated using techniques for the recovery of targeted deficiencies by FLP/FRT mediated excision of mapped transposon insertions [73], [74]. Overlapping deficiencies Df(2L)ppk1Aid(14334094–14383291) and Df(2L)ppk1MirB(14368856–14409711) are referred to in the text as DfA and DfB.

### Larval pupation height assays

Overall pupation height for a designated larval genotype was represented using a Pupation Height Index(PHI) calculated as ((# of pupae >3 cm) – (# of pupae <3 cm))/total # of pupae. Graphs of PHI, therefore, display a preference for higher pupation height as an upward deflection and preference for lower pupation height as a downward deflection.

Eggs were collected from 4 hour collections and seeded 50 eggs/vial onto the cut surface of fresh standard cornmeal-yeast-agar medium. After growth at 25°C(50% humidity) for three days, resulting larvae of the appropriate genotype were manually picked and seeded onto the cut surface of fresh vials containing fresh standard cornmeal-yeast-agar medium cut to a uniform height of 1.5 cm using a custom spatula. This transfer to fresh cut food vials within hours of food exit served to limit the influence of any phenotypic differences in food liquefaction prior to wandering stage. Vials were plugged with a 1 cm deep foam plug and incubated under constant light conditions to equalize the influence of light upon pupation height behavior. Seeded larvae were incubated at 25°C(50% humidity) until 96 h After Egg Laying(AEL) when they were shifted to an incubator at 29°C at indicated variations of humidity where they remained until the completion of pupation.

In order to make light and temperature exposure as uniform as possible for vials at any position within the rack, racks were placed upon a slowly rotating tray(2.5 Hz) for the duration of pupation. The temperature shift to 29°C was carried out to inactivate the temperature-sensitive Shi[ts1] and GAL80^ts^ proteins during larval wandering behavior. However, since the temperature shift itself can influence pupation height behavior, all vials were exposed to the same temperature shift protocol to allow direct comparison of results.

Once pupation was complete, vials were scored by counting the number of pupae found on the vial sides in 1 cm zones. Lethal desiccated larvae were identified both by their distinct morphology and by failure to undergo morphogenesis. Data was accumulated in Microsoft Excel and used to calculate PHI values for designated genotypes. Statistical analysis was carried out using Prism software to perform one-way ANOVA analysis with a Tukey posttest.

### Larval surface preference assay

Larvae of the appropriate genotype were seeded and allowed to grow following the same protocols and temperature shift paradigms described for pupation height behavior above. At the time of the temperature shift to 29°C, a 1.5×6 cm strip of Whatman #1 filter paper was inserted through the food to the bottom of the vial to stand vertically in the middle of the vial and wick moisture up from the food media. During wandering behavior, larvae had the ability to make a choice for pupation on the moistened filter paper or the dry glass sides of the vial. Once pupation was complete, a surface choice preference index was calculated by counting the number of pupae on the filter paper and the number on the glass sides of the vial. To compensate for the 2 dimensional surface contact distance of paper vs glass, the number of pupae on paper was multiplied by a factor of 2.3 to represent the relative difference between the 2×1.5 cm sides of the filter paper and the cirumference of the circular 25 mm glass vial. The resulting equation for calculating the Surface Choice Preference Index was: ((2.3×# on paper) - # on glass))/Total # pupae.

### Larval horizontal dry surface locomotion assay

Larvae were assayed for locomotion distance on a dry flat surface under constant humidity levels to rule out behavioral contributions from gravity and humidity gradients within the vertical culture vial. Wandering stage larvae of the appropriate genotype were picked from the side of vials and briefly rinsed in dH_2_O before being placed in groups of five on a 0.5 mm diameter circular filter(Whatman #1) that was uniformly wetted with 7.5 µl H_2_O. The filter was placed in the middle of a dry Pyrex glass dish at the center of a series of concentric circles differing in diameter by 1 cm. A plug of yeast/agar/cornmeal fly food was placed in one corner of the dish as an attractive odorant. The glass dish was placed within a sealed plexiglas chamber to allow temperature and humidity to be uniformly controlled. All experiments were performed at 20°C. This temperature was previously shown to be the preferred temp for wandering stage larvae [75]. Each group of larvae was allowed to crawl freely for 5 min at the end of which individual larvae were scored for their final position within the array of concentric circles. A higher score indicates increased distance of movement away from the wetted filter onto the dry glass surface. Statistical analysis was carried out using Prism software to perform one-way ANOVA analysis with a Tukey posttest.
